# Comparative transcriptome analysis of resistant and susceptible watermelon genotypes reveals the role of RNAi, callose, proteinase, and cell wall in squash vein yellowing virus resistance

**DOI:** 10.3389/fpls.2024.1426647

**Published:** 2024-08-02

**Authors:** Rahul Kumar, Bidisha Chanda, Scott Adkins, Chandrasekar S. Kousik

**Affiliations:** ^1^ Agricultural Research Service (USDA-ARS), U.S. Vegetable Laboratory (USVL), United States Department of Agriculture, Charleston, SC, United States; ^2^ ORISE participant, USVL, USDA-ARS, Charleston, SC, United States; ^3^ U.S. Horticultural Research Laboratory, USDA-ARS, Fort Pierce, FL, United States

**Keywords:** watermelon, SqVYV, WVD, transcriptome, RNAi, proteinase, callose

## Abstract

Watermelon (*Citrullus lanatus*) is the third largest fruit crop in the world in term of production. However, it is susceptible to several viruses. Watermelon vine decline (WVD), caused by whitefly-transmitted squash vein yellowing virus (SqVYV), is a disease that has caused over $60 million in losses in the US and continues to occur regularly in southeastern states. Understanding the molecular mechanisms underlying resistance to SqVYV is important for effective disease management. A time-course transcriptomic analysis was conducted on resistant (392291-VDR) and susceptible (Crimson Sweet) watermelon genotypes inoculated with SqVYV. Significantly higher levels of SqVYV were observed over time in the susceptible compared to the resistant genotype. The plasmodesmata callose binding protein (*PDCB*) gene, which is responsible for increased callose deposition in the plasmodesmata, was more highly expressed in the resistant genotype than in the susceptible genotype before and after inoculation, suggesting the inhibition of cell-to-cell movement of SqVYV. The potential role of the RNA interference (RNAi) pathway was observed in the resistant genotype based on differential expression of eukaryotic initiation factor (*eIF*), translin, DICER, ribosome inactivating proteins, RNA-dependent RNA polymerase (*RDR*), and Argonaute (*AGO*) genes after inoculation. The significant differential expression of hormone-related genes, including those involved in the ethylene, jasmonic acid, auxin, cytokinin, gibberellin, and salicylic acid signaling pathways, was observed, emphasizing their regulatory roles in the defense response. Genes regulating pectin metabolism, cellulose synthesis, cell growth and development, xenobiotic metabolism, and lignin biosynthesis were overexpressed in the susceptible genotype, suggesting that alterations in cell wall integrity and growth processes result in disease symptom development. These findings will be helpful for further functional studies and the development of SqVYV-resistant watermelon cultivars.

## Introduction

Watermelon (*Citrullus lanatus*, 2N = 22), is a member of the family Cucurbitaceae and the world’s third largest fruit crop in term of production. It is a globally cultivated cash crop renowned for its health-promoting compounds such as lycopene and citrulline (Kousik et al., 2015b; Maoto et al., 2019; Zamuz et al., 2021). In the United States, watermelon cultivation spans 96,500 acres, with an estimated value of $748 million (USDA, 2023). Nearly 80% of all U.S. watermelon production occurs in four states: Florida, Georgia, Texas, and California. Squash vein yellowing virus (SqVYV), the causal agent of watermelon vine decline (WVD), is one of the most important viral pathogens affecting watermelon crops in the southeastern United States, especially Florida (Huber, 2006; Roberts et al., 2008; Kousik et al., 2010; Kousik et al., 2012b; Adkins et al., 2013). Recognizing the severity of the situation, the National Watermelon Association identified WVD as a critical research priority (Morrissey, 2006; Kousik et al., 2016). This disease occurs during the spring and fall growing seasons in Florida and is characterized by a severe and sudden decline in watermelon vines and foliage as the crop approaches harvest or soon after the first harvest (Adkins et al., 2007; Kousik et al., 2012b; Webster et al., 2013). The symptoms include yellowing, scorched or brown leaves, defoliation, and wilting of the vines, leading to the rapid collapse of mature plants. In some fields, the incidence of WVD can increase from 10% to 80% within a week, indicating rapid disease progression (Kousik et al., 2012b). Fruits from affected plants are generally unmarketable and exhibit symptoms of internal rind necrosis and flesh decay, despite their external appearance being normal (Huber, 2006; Adkins et al., 2007; Roberts et al., 2008; Kousik et al., 2012b).

SqVYV is a potyvirus of the genus *Ipomovirus* (Adkins et al., 2007; Roberts et al., 2008). SqVYV was first identified in squash plants but has gained recognition for its devastating impact on watermelon. SqVYV is transmitted by whiteflies (*Bemisia tabaci*) in the agro-ecosytem (Webb et al., 2012) although experimentally SqVYV can also be mechanically transmitted (Adkins et al., 2007; Kousik et al., 2009; Webster et al., 2013). SqVYV is now widespread in the USA (Egel and Adkins, 2007; Adkins et al., 2011; Webster and Adkins, 2012; Batuman et al., 2015). In Florida, existing management strategies for WVD focus on eliminating reservoir host plants including cucurbit weeds and volunteer plants (Adkins et al., 2008; Kousik et al., 2012b). Though theoretically sound, complete eradication of the virus reservoirs is challenging and extremely difficult in practice. Additionally, whitefly populations are managed through insecticide application and the use of silver plastic mulch (Kousik et al., 2010; Kousik et al., 2015a). In addition, there has been an alarming increase in the number of whitefly populations resistant to insecticides, particularly neonicotinoids (Patra and Hath, 2022). Therefore, the development of SqVYV-resistant cultivars is one of the best options for managing this devastating disease. Our laboratory has identified sources of resistance to SqVYV in watermelon germplasm and developed 392291-VDR, a resistant watermelon germplasm (Kousik et al., 2009; Kousik et al., 2012a).

Plants have developed a series of defense mechanisms against pathogen attacks during their coevolution (Métraux et al., 2009; Qi et al., 2011; Tena, 2021). Recognition and signaling pathways are central to the defense against viral infections in plants. Pattern recognition receptors (PRRs) enable plants to detect conserved viral components known as pathogen-associated molecular patterns (PAMPs), initiating a cascade of signaling events that activate defense responses (Jones and Dangl, 2006). Signaling pathways, such as the salicylic acid (SA), jasmonic acid (JA), and ethylene (ET) pathways, play pivotal roles in mediating antiviral defense responses by regulating the expression of defense genes and coordinating various defense mechanisms (Pandey and Baldwin, 2007; Pieterse et al., 2012). An essential mechanism employed by plants to combat viral infections is RNA silencing, also known as RNA interference (RNAi) (Ding and Voinnet, 2007; Hung and Slotkin, 2021). This conserved pathway involves the production of small RNA molecules, including small interfering RNAs (siRNAs) and microRNAs (miRNAs), which target and degrade viral RNA (Ding and Voinnet, 2007; Hung and Slotkin, 2021). RNA silencing acts as a potent antiviral defense mechanism, suppressing viral replication and spread. Another critical component of plant defense against viral diseases is the presence of resistance (R) genes. These genes encode intracellular immune receptors that directly or indirectly recognize viral effector molecules. Recognition of effectors leads to the activation of effector-triggered immunity (ETI), which often manifests as a hypersensitive response (HR) characterized by localized cell death at the infection site (Jones and Dangl, 2006). In collaboration with other defense pathways, such as SA-mediated signaling, R genes confer resistance against specific strains or types of viruses, enhancing plant immunity (Dangl et al., 2013). Plants also deploy cellular structural barriers to defend against pathogens, hindering their initial entry and subsequent cell-to-cell spread. The polysaccharide callose is one such barrier made up primarily of β-1,3-glucan chains that play important roles in the defense against biotic stresses, including viral infections (Li et al., 2012; Wang et al., 2021; Zhang et al., 2022). In addition, antioxidant mechanism also helps plants in combating stresses (Ding et al., 2020; Rana et al., 2021, 2022; Kumar et al., 2023; Chugh et al., 2024). The intricate interplay of these defense mechanisms provides plants with layered and dynamic resistance against viral diseases.

Since WVD caused by SqVYV is an important disease, understanding the molecular basis of resistance could be useful for developing vine decline resistant watermelon varieties. To date, no transcriptomic study of vine decline disease has been reported in watermelon. To address these knowledge gaps, 392291-VDR (resistant) developed by USDA ARS (Kousik et al., 2012a) and the commercial cultivar Crimson Sweet (susceptible) watermelon genotypes were used to understand the molecular basis of WVD resistance. The findings of this study enhance our understanding of WVD resistance mechanisms and provide valuable insights for the development of targeted approaches for watermelon disease management.

## Materials and methods

### Plant materials and virus inoculation

Two watermelon genotypes, 392291-VDR (Resistant) and Crimson Sweet (Susceptible), were used in this study. The SqVYV-resistant watermelon germplasm, 392291-VDR (*Citrullus lanatus*) was identified and developed in our lab based on phenotyping 218 plant introductions (PI) by mechanical inoculation with SqVYV (Kousik et al., 2009; 2012a). Seeds of the cultivar Crimson Sweet, developed by C.V. Hall through crossing ‘Peacock’ and ‘Chubby Gray’s’ at Kansas State University in 1964, were obtained from Willhite Seeds (Poolville, TX). The original squash isolate of SqVYV was obtained from Hillsborough County, FL, as described previously (Adkins et al., 2007, 2008) and routinely maintained in Prelude II squash plants (*Cucurbita pepo*, Seminis Seeds, Oxnard, CA). The virus inoculum was prepared by homogenizing infected plant tissue, including leaves, cotyledons, and hypocotyls, in 20 mM sodium phosphate buffer (pH 7.0) containing 0.1% (wt/vol) sodium sulfite and 1% (wt/vol) celite following the protocol described by Adkins et al. (2008). Mechanical inoculation of the resistant and susceptible genotypes was carried out by gently rubbing the inoculum onto the cotyledons and first true leaves of 4-week-old plants using cheese cloth. After inoculation, the plants were placed in a walk-in Percival (https://www.percival-scientific.com/) growth chamber at 28°C with 12 h day/night light cycles. The control and zero-time-point plants were mock-inoculated with sodium phosphate buffer only. The plants were then monitored every day for vine decline symptom expression.

### RNA extraction, cDNA library construction, and Illumina sequencing

Plant tissues including hypocotyl and true leaves were collected from both the 392291-VDR and Crimson Sweet genotypes at four time points: 0 (before inoculation), 5, 10, and 15 days after inoculation. Plant tissues were frozen in liquid nitrogen immediately after harvesting and stored at -80°C. Total RNA was isolated from frozen plant tissue using TRIzol reagent (Invitrogen, Carlsbad, CA, USA) following the manufacturer’s instructions. RNA purification steps and on-column DNase digestion were performed using the QIAGEN RNeasy Mini Kit as suggested by the manufacturer (QIAGEN, Hilden, Germany). Paired-end sequencing was performed using a NovaSeq 6000 SP v1.5 200 cycle sequencing (2 × 100 bp) instrument. Three biological replicates of each genotype (392291-VDR and Crimson Sweet) per time point were used for 100 bp paired-end RNA-seq. The original RNA-seq data has been submitted to the National Center for Biotechnology Information (NCBI) and can be accessed under the bioproject number PRJNA1086032.

### Processing and mapping of Illumina reads

Transcriptome analysis was done by Novogene. The quality of the raw sequencing data was assessed using FastQC (fastqc/0.12.1 version) (Andrews, 2010) to ensure high data quality. Subsequently, Trimmomatic (trimmomatic/0.39 version) was used to remove adapter sequences and low-quality reads, resulting in a set of high-quality reads for downstream analysis (Bolger et al., 2014). Low-quality reads with a minimum Phred quality score of less than 35 were trimmed from both ends. To ensure a minimum length requirement, reads with 30 or more nucleotides (for each pair) were retained. The high-quality reads were then mapped to the Charleston gray watermelon reference genome (Wu et al., 2019) (http://cucurbitgenomics.org/organism/4) using the mem algorithm from the Burrows−Wheeler aligner (bwa-mem2/2.1 version) (Li and Durbin, 2009). edgeR (edgeR_4.0.16 version) was used to identify differentially expressed genes (Robinson et al., 2010). Genes with a log2FC > 2 and < −2 were considered up- and downregulated, respectively. A false discovery rate (FDR) ratio threshold of less than 0.05 was used to filter out the most significantly differentially expressed genes (DEGs).

### Gene annotation, classification of DEGs into functional categories, and KEGG analysis

The Gene Ontology (GO) classifications of all DEGs/transcripts were categorized into broader GO classes using the GO enrichment and GO gene classification tools available in the Cucurbit Genomics Database (https://cucurbitgenomics.org/pwyenrich) and Blast2GO server (https://www.blast2go.com/). KEGG pathway analysis was performed using the KOBAS 3 database (https://kobas.cbi.pku.edu.cn/kobas3).

### Quantitative real-time PCR validation of select differentially expressed genes

Eight important DEGs were confirmed by qRT-PCR, and the sequences of the primers used are listed in **Table 1**. Three biological replicates were used for the qRT−PCR analysis. cDNA was synthesized using the iScript™ cDNA Synthesis Kit (Bio-Rad Laboratories, Richmond, CA, US). Subsequently, these cDNA products were used in qRT−PCR assays with SYBR Green I Master Mix (2× concentration). The reactions were carried out on a LightCycler 480 Instrument II (Roche, Basel, Switzerland) in 96-well plates. Each well had a reaction solution volume of 20 μL. For normalization purposes, the native actin gene was used as the internal control, as suggested in prior studies (Mandal et al., 2018). The expression levels were standardized against the actin gene expression for each respective sample using the comparative Ct method (2^−ΔΔCt^) (Livak and Schmittgen, 2001).

**Table 1:** Primers used for the validation of DEGs.

Gene Name	Forward (5’-3’)	Reverse (5’-3’)
Actin	TCAGCAACTGGGATGATATGG	TGAGAGGAGCTTCGGTAAGA
Plasmodesmata callose binding protein (*PDCB*),	ACGACGAATCCAGGAATGAC	CTGCCAAAAGCAAGTTCCTC
Eukaryotic initiation factor 4 (*EIF4*)	GGACTACGACGAGGAGCTTG	TACCGCCATTCTGATCCTTC
Dicer like protein 4 (*DLP4*)	GTGAGACCAAGTGCAGCAAA	AAGCCGCTGTCAACTAGGAA
Argonaute (*AGO*)	GGATCCAACCGTGAAGAGAA	GATCCAAGGATGGGTCAATG
Limonene synthase (*LMS*)	TTCCACGATGGAGGAGAGAC	TCCTGAGGGATGTGATAGCC
Ethylene-responsive proteinase inhibitor 1 (*ERR1*)	TGGCCGGAACTTGTTGGAAT	GTCACTCCACTTCCAGCCAA
Viral movement protein (*VMP*)	GAGCAGCTTCCTAAAGGGACA	CTCTCAAGCAAAGCATCTGGC
non-specific lipid transfer proteins (*nsLTP*)	CATGACGTGCAACCAAGTGG	TCACACCGTAGCAACAGGTC
SqVYV	GTGACAACAACCGTCGCG	TCCTCCCTTGCAGCTCAATG

## Results

### 392291-VDR genotype showed resistance to SqVYV

**Figure 1:**
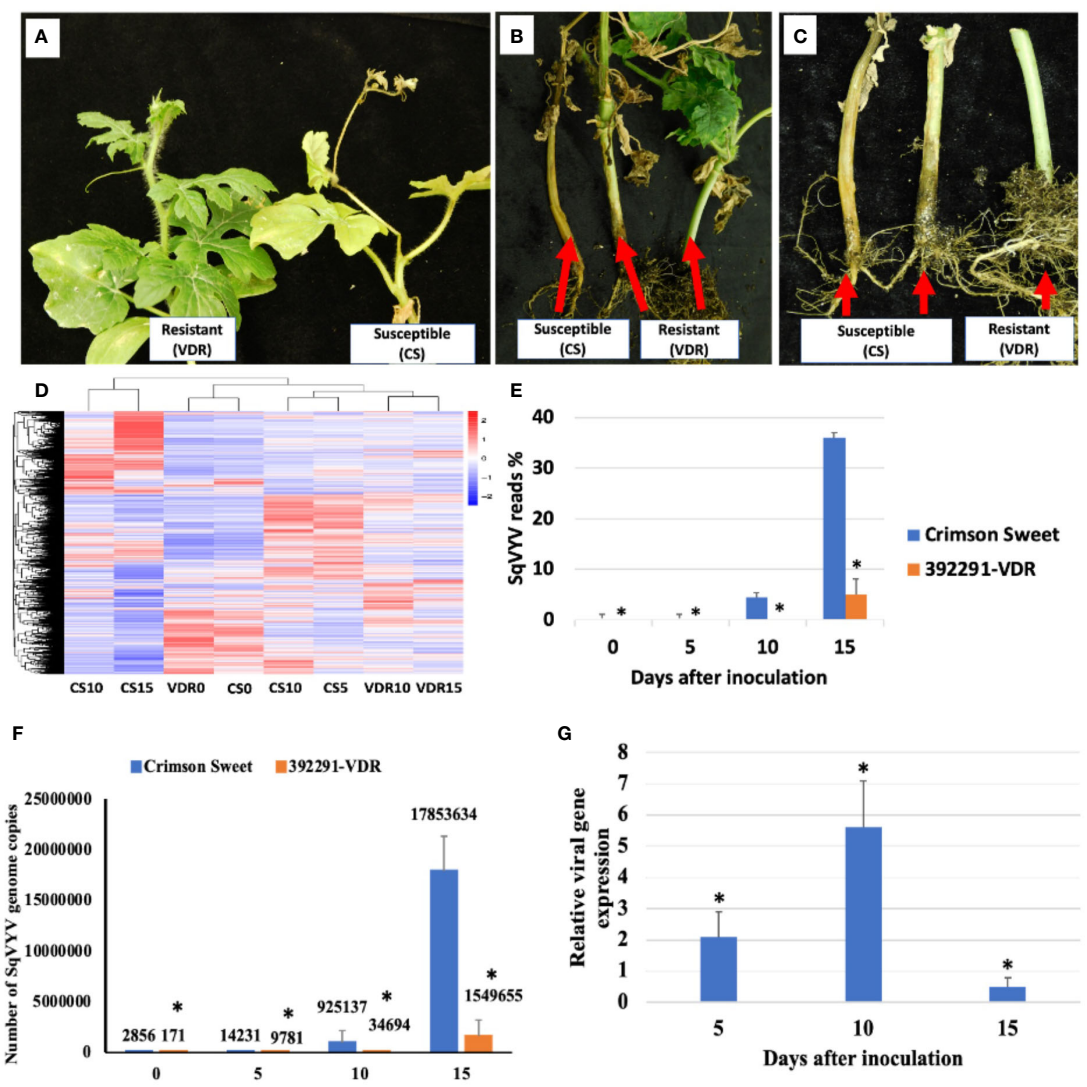
Assessment of phenotype and viral loads following SqVYV inoculation. **(A–C)** 392291-VDR (VDR) genotype showed SqVYV resistance compared to Crimson Sweet (CS) after 15 days of the inoculation. Crimson Sweet showed a typical watermelon vine decline (WVD) symptoms including yellowing, defoliation **(A)**, stem necrosis **(B)**, reduced growth, and collapse of hypocotyl **(C)**. **(D)** Heatmaps showing hierarchical cluster analysis of differentially expressed genes at 0, 5, 10, and 15 days post inoculation. **(E, F)** The transcriptome data were mapped to the SqVYV genome, and the aggregate number of reads was determined. The viral load in both the resistant and susceptible genotypes increased after inoculation; however, the viral load in the resistant genotype remained significantly lower than that in the susceptible genotype. **(G)** Relative gene expression analysis of SqVYV using qRT-PCR. Higher SqVYV gene expression was observed in susceptible genotype compared to resistant after 5 and 10 dpi. However, after 15 dpi, the gene expression decreased in susceptible genotype compared to resistant, possibly due to tissue death. Asterisks indicate statistically significant differences compared to susceptible control (student's t-test): *p < 0.05.

Fifteen days post-inoculation (dpi), all plants were scored for the WVD symptoms (**Figures 1A–C**). Crimson Sweet plants showed characteristic symptoms of WVD, which included yellowing, scorched or brown leaves, defoliation, and wilting of the vines. Conversely, 392291-VDR plants exhibited resistance to the disease, showing significantly milder symptoms than Crimson Sweet (**Figures 1A–C**). To calculate the number of viral copies in the plants, the transcriptome data were mapped to the SqVYV genome. The percent reads for each sample were determined based on the overall count of reads in the samples. Following inoculation, both the resistant and susceptible genotypes exhibited increased viral loads (reads) (**Figures 1D, E**). However, the susceptible genotypes had a significantly much higher number of mapped reads compared to the resistant genotypes. After 5, 10, and 15 dpi, the susceptible genotypes accounted for 0.026% (14231 copies), 4.4% (925137 copies), and 36% (17853634 copies) of the total reads, respectively (**Figures 1E, F**). In comparison, the resistant genotypes produced averages of 0.021% (9781 copies), 0.08% (34694 copies), and 4.9% (1549655 copies) at the same time intervals. Relative gene expression analysis of SqVYV was performed using qRT-PCR. A 2-fold and 5.5-fold higher expression of the SqVYV gene was observed in the susceptible genotype compared to the resistant genotype after 5 and 10 dpi, respectively (**Figure 1G**). However, after 15 dpi, the gene expression decreased by 50% in the susceptible genotype compared to the resistant genotype, possibly due to tissue death.

### Evaluation of RNA-seq data

The genome-wide gene expression profile was obtained through RNA-seq analysis of resistant and susceptible genotypes before and after inoculation. RNA-seq analysis was conducted at four time points: 0 (before inoculation), 5, 10, and 15 days. The resulting RNA-seq libraries generated 19.7 to 42.8 million reads (**Table 2**). The highest number of reads (46.40 million) was observed for the VDR-15 (392291-VDR at 15 dpi), while the lowest number of reads (41.28 million) was observed for the VDR-10 (392291-VDR at 10 dpi). Most of these reads (44% to 97%) were successfully mapped to the watermelon reference genome, resulting in transcriptome coverage ranging from 19X to 25X (**Table 2**). Among the total mapped reads, VDR-0 (392291-VDR at 0) has highest mapping with 42.83 million reads, while CS-15 (Crimson Sweet at 15 dpi) had the lowest at 19.77 million. In terms of unique mapping, VDR-0 again had highest uniquely mapped reads with 41.08 million, with CS-15 having the lowest at 18.92 million. Considering the percentage of total mapping rates, VDR-0 had the highest percentage, at 96.94%. In contrast, CS-15 had the lowest percentage, at 43.45%. VDR-0 had the highest unique mapping rate of 92.99%, and CS-15 had the lowest unique mapping rate of 41.58%. Multiple mapping reads ranged from a maximum of 1.75 million in VDR-0 to a minimum of 0.85 million in CS-15. Multiple mapping rates varied moderately across samples, with the highest being 3.95% for VDR-0 and the lowest being 1.87% for CS-15.

**Table 2:** RNA-seq read statistics of 392291-VDR (VDR) and Crimson Sweet (CS) before and after SqVYV inoculation.

Sequence	VDR0	VDR5	VDR10	VDR15	CS0	CS5	CS10	CS15
**Total reads (M)**	44.18	44.58	41.28	46.40	42.45	42.68	44.83	45.88
**Total mapped reads (M)**	42.83	38.28	28.43	33.40	36.49	37.15	33.53	19.77
**Uniquely mapped reads (M)**	41.08	36.91	27.43	32.20	35.0	35.85	32.18	18.92
**Multiple mapped reads (M)**	1.75	1.37	1.00	1.20	1.47	1.31	1.31	0.85
**Total mapping rate (%)**	96.94	85.77	69.30	72.18	86.38	87.33	74.62	43.45
**Uniquely mapping rate (%)**	92.99	82.70	66.86	69.58	82.90	84.26	71.63	41.58
**Multiple mapping rate (%)**	3.95	3.07	2.44	2.60	3.48	3.07	2.99	1.87

Total reads (M) - The total number of sequencing reads generated for each sample in millions. Total mapped reads (M) - The number of reads that were successfully aligned to the reference genome. Uniquely mapped reads (M) - The subset of mapped reads that aligned to a unique location in the reference. Multiple mapped reads (M) - The subset of mapped reads that aligned to multiple locations in the reference. Total mapping rate (%) - The percentage of total reads that were successfully mapped to the reference. Uniquely mapping rate (%) - The percentage of total reads that uniquely mapped to a single location in the reference. Multiple mapping rate (%) - The percentage of total reads that mapped to multiple locations in the reference. VRD0, VRD5, VRD10, and VRD15 denote the observations for 392291-VDR at 0, 5, 10, and 15 dpi, respectively. Similarly, CS0, CS5, CS10, and CS15 represent the data points for Crimson Sweet at 0, 5, 10, and 15 dpi, respectively.

### Analysis of differentially expressed genes

**Figure 2:**
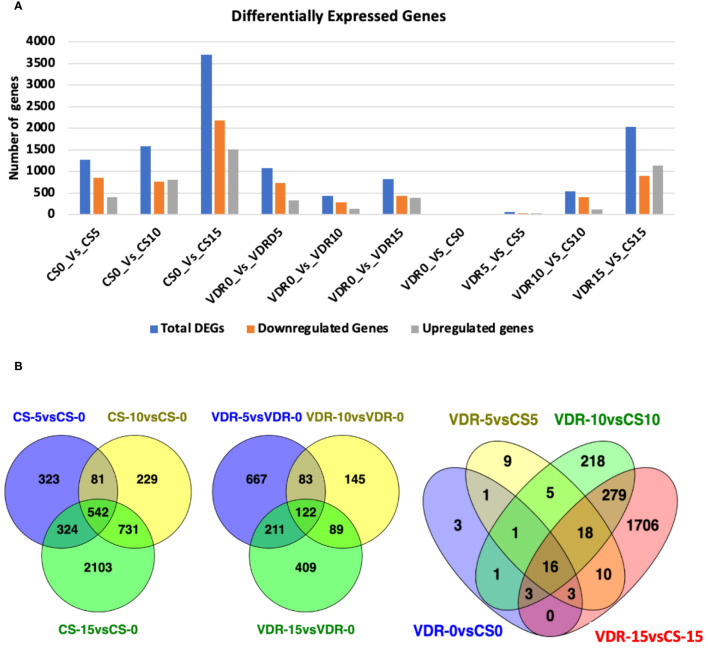
Numbers of differentially expressed genes (DEGs) and Venn diagram of DEGs in the transcriptomes of the VDR and CS at different time points. **(A)** Numbers of DEGs in Crimson Sweet (CS) and 392291-VDR (VDR) at various time points before and after inoculation with SqVYV. Numbers of DEGs were increased between VDR and CS genotypes after inoculation with the time. A higher number of DEGs were observed in CS compared VDR in response SqVYV inoculation. **(B)** All DEGs are grouped into different comparison groups represented by circles. The overlapping portions of the circles show the number of DEGs common to these groups.

Differentially expressed genes (DEGs) were identified by comparing treatments with a log2FC (fold change) greater than 2 and an adjusted p value (p-adj) at or less than 0.05 (**Supplementary Table 1**). SqVYV infection induced dramatic changes in cellular and metabolic processes. In the Crimson Sweet genotype, 1270 DEGs (412 upregulated and 858 downregulated) were identified at the early infection phase (5 dpi) compared to 0 dpi (**Figure 2A**). There were 1583 (814 upregulated and 769 downregulated) DEGs at 10 dpi compared to 0 dpi. After 15 dpi, there were a total of 3700 DEGs, with 1516 upregulated and 2184 downregulated genes. In the resistant genotype, 392291-VDR, there were 1083 DEGs during the early stages of infection (5 dpi) compared to 0 dpi, comprising 339 upregulated and 744 downregulated genes (**Figure 2A**). Interestingly, after 10 dpi, there were a total of 439 DEGs, consisting of 148 upregulated and 291 downregulated genes. During the late stages of infection (15 dpi), there were 831 DEGs, comprising 394 upregulated and 437 downregulated genes.

There were 27 DEGs (13 upregulated and 14 downregulated) between 392291-VDR and Crimson Sweet before infection (**Figure 2A**). At 5 dpi, 63 DEGs (29 upregulated and 34 downregulated) were observed in the 392291-VDR compared to the Crimson Sweet. A total of 541 DEGs, comprising 127 upregulated and 414 downregulated genes, were observed in 392291-VDR compared to Crimson Sweet at 10 dpi. At 15 dpi, 2035 DEGs (1141 upregulated and 894 downregulated) were observed in 392291-VDR compared to Crimson Sweet. These results indicate that SqVYV infection caused drastic changes in both genotypes, but expectedly, more changes were observed in the susceptible genotype.

A common DEGs were identified at different point of inoculation in both resistant and susceptible genotypes (**Figure 2B**). 542 common DEGs were identified at 5, 10, and 15 dpi compared to 0 dpi in Crimson Sweet. Similarly, 122 common DEGs were found at the same time points in 392291-VDR compared to 0 dpi. Before and after SqVYV inoculation, a total of 34 DEGs were identified between Crimson Sweet and 392291-VDR, with 18 DEGs identified after inoculation, suggesting their potential significance in SqVYV resistance (**Figure 2B**).

### Functional annotation and pathway enrichment analysis of DEGs

**Figure 3:**
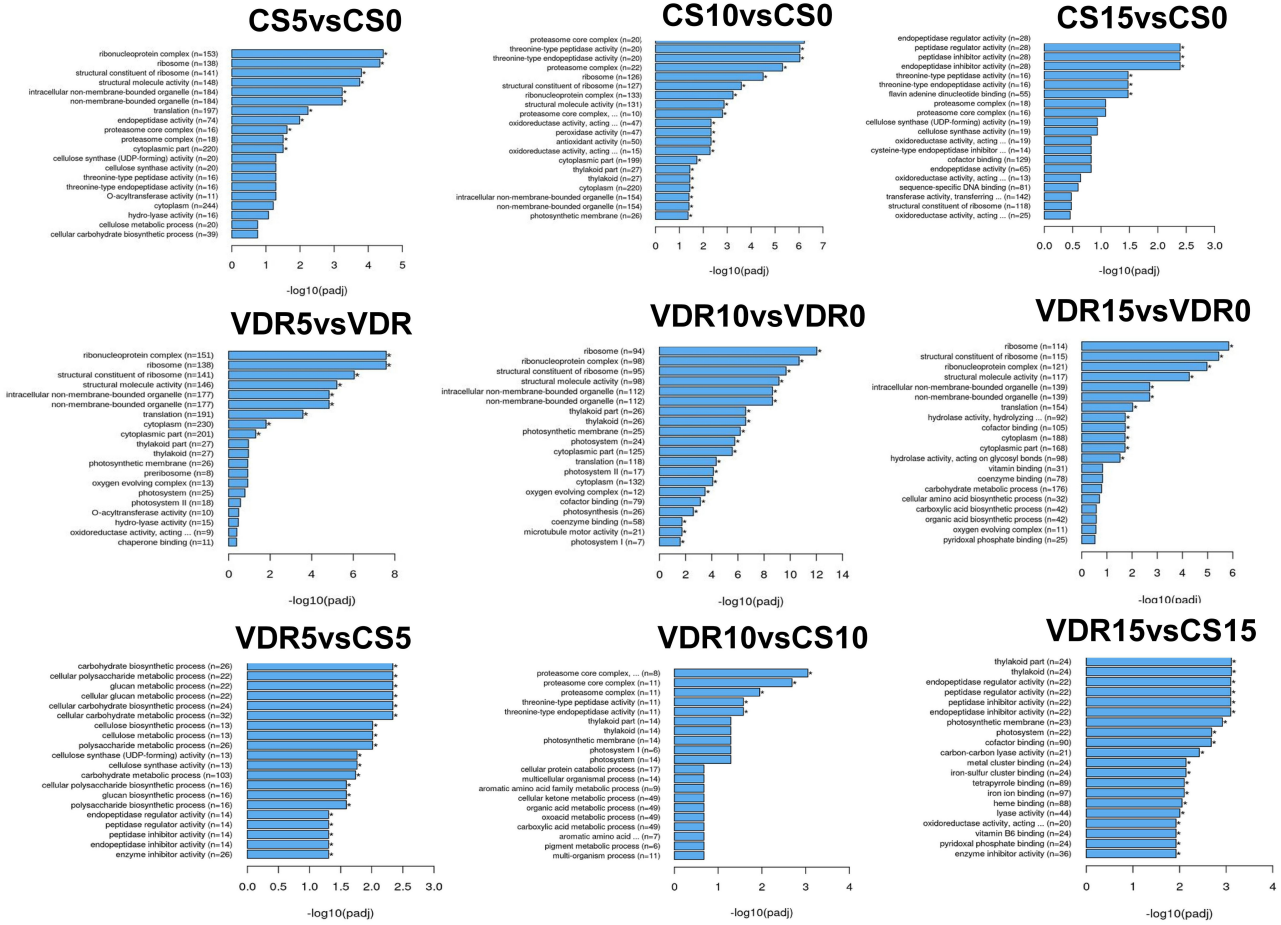
Gene Ontology (GO) functional enrichment analysis of the DEGs identified. DEGs were identified before and after inoculation in the 392291-VDR (VDR) and Crimson Sweet (CS) genotypes. *Asterisk means DEGs were significantly enriched in this GO term.

Gene Ontology (GO) enrichment analysis of the identified DEGs was performed to provide insights into the biological processes that play a role in resistance and a common response to SqVYV infection. During the infection stages (5 and 10 dpi), the GO functional categories for Crimson Sweet and 392291-VDR displayed some similarities when compared with those at 0 dpi (**Figure 3**). After 5 and 10 dpi, ribonucleoprotein, ribosome, cytoplasm, and translation related genes were differentially expressed in both genotypes. However, at 15 dpi, peptide related genes were predominantly differentially expressed in the susceptible genotype. On the other hand, in the resistant genotype, genes related to ribonucleoprotein, ribosome, cytoplasm and translation were differentially expressed at 15 dpi (**Figure 3**).

### Virus replication inhibitor genes differentially expressed between resistant and susceptible genotypes

**Figure 4:**
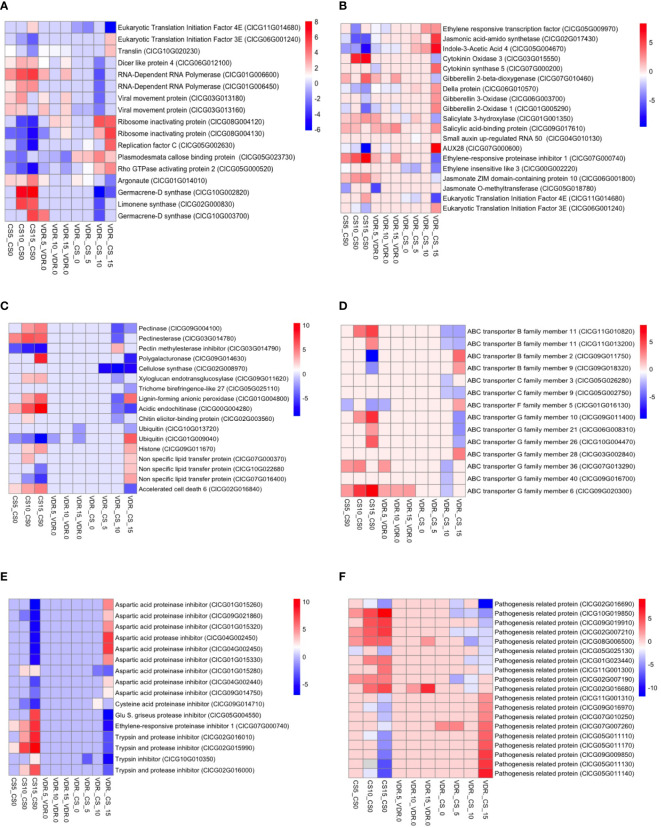
Transcriptome heatmap for DEGs in Crimson Sweet (CS) and 392291-VDR (VDR) post-inoculation. The heatmap shows the RNA-Seq transcriptome analysis results for 35 selected genes from Crimson Sweet (CS) and 392291-VDR (VDR) at 0, 5, 10, and 15 dpi. Each row corresponds to a specific gene, and columns represent ten different interactions. Upregulated genes are indicated in red, whereas downregulated genes are indicated in blue. The color gradient illustrates the log2 fold changes in gene expression, providing insights into the differential responses of the Crimson Sweet (susceptible) and 392291-VDR (resistant) genotypes to SqVYV infection. The heatmap categorizes genes into four groups: **(A)** Genes involved in disease resistance and plant-pathogen interaction **(B)** Genes involved in plant hormones, **(C)** Disease symptoms **(D)** ABS transporter, **(E)** Proteinase inhibitor, and **(F)** Pathogenesis related proteins.

The resistant genotypes exhibited altered expression of genes involved in RNA-related processes, including those involve in the inhibition of virus replication. Eukaryotic initiation factors (ClCG11G014680, ClCG06G001240), translin (ClCG10G020230), Dicer-like protein 4 (ClCG06G012100), RNA-dependent RNA polymerase (ClCG01G006600, ClCG01G006450), and Argonaute (ClCG01G014010) genes were differentially expressed between the resistant and susceptible genotype after inoculation (**Figure 4A**). These genes play crucial roles in RNA interference mediated viral disease resistance in plants. Eukaryotic initiation factor 4E (ClCG11G014680) was upregulated in the susceptible genotype compared to resistant after inoculation, while this gene expression did not change in the resistant cultivar. On the contrary, the eukaryotic initiation factor 3 E (ClCG06G001240) gene was overexpressed in the resistant cultivar compared to the susceptible cultivar after inoculation. Translin, an important component of the antiviral RNA interference (RNAi) pathway, was 4.7 times overexpressed in the resistant cultivar compared to the susceptible cultivar after 15 days of inoculation. Dicer like protein 4 (ClCG06G012100) and Argonaute (ClCG01G014010), an integral components of the antiviral RNA interference (RNAi) pathway, were upregulated in the susceptible cultivar after inoculation relative to the resistant cultivar (**Figure 4**). Similarly, the expression of RNA-dependent RNA polymerase ClCG01G006600 and ClCG01G006450 was upregulated 52 and 11 times in the susceptible cultivar at 10 dpi, respectively (**Figure 4A**). Ribosome-inactivating proteins are a class of enzymes found in various organisms, including plants and fungi. These proteins have the ability to inhibit protein synthesis by catalyzing the removal of adenine residues from the ribosomal RNA, thus rendering the ribosome unable to function properly. Ribosome-inactivating proteins confer resistance to viruses in plants. Two ribosome-inactivating proteins (ClCG08G004120, ClCG08G004130) were downregulated 2.5 to 5 times in the susceptible genotype after inoculation, while resistant genotype maintained expression level. Prior to inoculation, the expression of the plasmodesmata callose binding protein (*PDCB*) gene (ClCG05G023730), which plays a crucial role in inhibiting virus movement, was 4.5 times greater in the resistant genotype compared to the susceptible. At 5, 10, and 15 dpi, the expression levels in the resistant genotype were elevated by 4.9, 13.4, and 4.7 times, respectively, compared to the susceptible genotype. In the susceptible genotype, the expression of the *PDCB* gene at 5, 10, and 15 dpi was reduced 4, 5.2, and 7.9 times, respectively, compared to 0 dpi. Interestingly, in the resistant genotype, there were no significant changes in the expression levels after 5 and 10 dpi compared to 0 dpi. However, after 15 days the expression decreased to 4.4 times lower than the 0 dpi but was still 4.7 times greater than the susceptible genotype (**Figure 4A**).

### Role of pathogenesis related genes in disease resistance

Proteinase inhibitor, ubiquitin, histone, pathogenesis related proteins and ABC transporter genes were differentially expressed in both genotypes specially in susceptible after SqVYV inoculation (**Figures 4C–F**). Proteinase inhibitors play important roles in viral resistance in plants. Proteinase inhibitors can inhibit viral proteases, thereby disrupting viral replication and spread within the host plant. In this study, 16 proteinase inhibitor genes showed a differential expression in the susceptible genotypes after inoculation (**Figure 4E**). These genes included ClCG04G002450, ClCG10G010350, ClCG05G004550, ClCG02G015990, ClCG02G016010, ClCG02G016000, ClCG01G015280, ClCG09G014710, ClCG07G000740, ClCG09G014750, ClCG04G002440, ClCG09G021860, ClCG01G015320, ClCG01G015330, ClCG01G015260, and ClCG04G002450. Out of these 16 proteinase genes, 5 were upregulated and 11 downregulated. These proteinase inhibitors include aspartic acid proteinase inhibitor, ethylene-responsive proteinase inhibitor, cysteine acid proteinase inhibitor, aspartic acid proteinase inhibitor, trypsin and protease inhibitor, glu S. griseus protease inhibitor, trypsin inhibitor, and aspartic acid protease inhibitor. nsLTPs are small, cysteine-rich proteins and are known to have diverse roles in plant defense mechanisms, including defense against viral pathogens. 19 pathogenesis related proteins including ClCG01G023440, ClCG02G007190, ClCG02G007210, ClCG02G016680, ClCG02G016690, ClCG05G011110, ClCG05G011130, ClCG05G011140, ClCG05G011170, ClCG05G025130, ClCG07G007260, ClCG07G010250, ClCG08G006500, ClCG09G009850, ClCG09G016970, ClCG09G019910, ClCG10G019850, ClCG11G001300, and ClCG11G001310 were differentially expressed in susceptible genotype after inoculation (**Figure 4F**). Out of 19 genes, 9 upregulated and 10 downregulated. In the resistant genotype, only ClCG02G016690 and ClCG08G006500 genes were upregulated after inoculation and other genes did not significantly change.

Fourteen ATP-Binding Cassette (*ABC*) transporter genes (ClCG11G010820, ClCG11G013200, ClCG09G011750, ClCG09G018320, ClCG05G026280, ClCG05G002750, ClCG01G016130, ClCG09G011400, ClCG06G008310, ClCG10G004470, ClCG03G002840, ClCG07G013290, ClCG09G016700, ClCG09G020300) were differentially expressed between the resistant and susceptible genotypes after inoculation (**Figure 4D**). Of these 14 genes, 4 belong to the B family, 2 to the C family, 1 to the F family, and 7 to the G family. Among these 14 genes, 11 were upregulated, and 3 were downregulated in the susceptible genotype after inoculation. In the resistant genotypes, only ClCG01G016130, ClCG09G020300, and ClCG07G013290 genes differentially expressed after inoculation. Among the ubiquitin genes analyzed, ClCG10G013720 exhibited significant downregulation in the SqVYV-resistant watermelon genotype compared to susceptible cultivars. This downregulation suggests a potential role of ClCG10G013720 in the resistance mechanism against SqVYV infection. Conversely, ClCG01G009040 showed significant upregulation, indicating its potential involvement in the defense response to SqVYV. In the histone gene family, ClCG09G011670 displayed significant upregulation in the resistant watermelon genotype. We also observed the reduced expression of nsLTP genes in the susceptible genotypes after SqVYV inoculation including ClCG07G000370, ClCG10G022680, and ClCG07G016400. In addition, the accelerated cell death 6 (*ACD6*) gene (ClCG02G016840) was upregulated 4, 12, and 28 times in the susceptible genotype after 5, 10, and 15 dpi, respectively, compared to 0 dpi.

### Role of hormones and SqVYV resistance in watermelon

Viral infection altered the expression of hormone-related genes, including those involved in the ethylene, jasmonic acid, auxin, cytokinin, gibberellin, and salicylic acid signaling pathways, suggesting the roles of these genes in defense responses against SqVYV (**Figure 4B**). Jasmonic acid-amino synthetase (ClCG02G017430) exhibited a 39.4 times downregulation after 10 dpi compared to 0 dpi in the susceptible genotype. The jasmonate ZIM domain-containing protein 10 (ClCG06G001800) gene was 4.9 and 7 times upregulated after 10 and 15 dpi, respectively, compared to 0 dpi in the susceptible genotype. Jasmonate O-methyltransferase (ClCG05G018780) showed 11 times downregulation after 5 dpi compared to 0 dpi in the susceptible genotype. In contrast, the expression levels of each of these genes did not change in the resistant genotype after inoculation. The expression of indoleacetic acid 4 (*IAA4*) (ClCG05G004670), a gene involved in auxin signaling pathways, was 10.6, 11.4, and 223 times downregulated after 5, 10, and 15 dpi, respectively, compared to 0 dpi in the susceptible genotype. In the resistant genotype, the expression of this gene decreased about 4.3 times at 5 dpi and maintained expression level at 10 and 15 dpi.

The expression level of the cytokinin oxidases/dehydrogenases 3 (*CKX3*) (ClCG03G015550) gene was elevated 69 and 137 times at 10 and 15 dpi, respectively, in the susceptible genotype compared at 0 dpi, while no significant change was observed in the resistant genotype. In the susceptible genotype, gibberellin 3-oxidase (*GA3ox*) (ClCG06G003700) and gibberellin 20-oxidase (*GA20ox*) (ClCG01G005290) were 4.6 and 32 times downregulated, respectively, after 15 dpi compared to 0 dpi, while della protein (ClCG06G010570) was downregulated 6.5 and 13 times at 10 and 15 dpi. In the resistant genotype, *GA20ox* gene was 4.3 times downregulated after 5 dpi compared to 0 dpi, while no significant changes were observed in *GA3ox* and della genes. The negative regulator of gibberellic acid biosynthesis pathway, Gibberellin 2-beta-dioxygenase (*GA2ox*) (ClCG07G010460) was 16 and 4 times upregulated in susceptible and resistant genotypes, respectively, after 15 dpi compared to 0 dpi. Ethylene responsive transcription factor (ClCG05G009970) was 18.4 times upregulated in the susceptible genotype after 15 dpi compared to 0 dpi. The expression level of the ethylene-responsive proteinase inhibitor 1 (ClCG07G000740) gene was elevated 6.5, 21, and 256 times at 5, 10, and 15 dpi, respectively, in the susceptible genotype compared to 0 dpi. No significant change was observed in both genes in the resistant genotype. The resistant genotype exhibited 4.5, 7.8, 17, and 20 times lower expression of the ethylene-insensitive-like 3 (ClCG00G002220) gene at 0, 5, 10, and 15 dpi, respectively, compared to the susceptible genotype. After 15 dpi, salicylate 3-hydroxylase (ClCG01G001350) was 4.9 times upregulated in the susceptible genotype and salicylic acid-binding protein (ClCG09G017610) was 6.5 times upregulated in the resistant genotype, compared to 0 dpi. Auxin-induced protein (AUX28) (ClCG07G000600) was 776 times downregulated in the susceptible genotype after 15 dpi compared to 0 dpi, and no significant change was observed in the resistant genotype.

### Identification of DEGs involved in disease symptom development

The susceptible genotypes showed DEGs associated with plant cell wall modification, including those related to pectin, polygalacturonase, lignin, and chitin after SqVYV inoculation (**Figure 4C**). Pectinase (ClCG09G004100), pectinesterase (ClCG03G014780), pectin methylesterase inhibitor (ClCG03G014790), and polygalacturonase (ClCG09G014630), which play important roles in pectin metabolism and modification, were overexpressed in the susceptible genotypes after infection. Furthermore, cellulose synthase (ClCG02G008970), involved in cellulose synthesis and cell wall architecture, showed higher expression in the susceptible genotypes. The expression levels of xyloglucan endotransglucosylase (ClCG09G011620) and trichome birefringence-like 27 (ClCG05G025110) genes associated with cell elongation and development were also upregulated in the susceptible genotypes. Furthermore, genes associated with lignin and chitin biosynthesis showed overexpression in the susceptible genotypes. Lignin-forming anionic peroxidase (ClCG01G004800), acidic endochitinase (ClCG00G004280), and chitin elicitor-binding protein (ClCG02G003560) genes were among those upregulated.

### SqVYV infection upregulates the watermelon terpenoid pathway

Terpenes are a diverse class of volatile organic compounds produced by plants (Glaeske and Boehlke, 2002). Terpenoid pathway genes were highly upregulated in the susceptible variety after SqVYV inoculation (**Figure 4A**). Terpenoid biosynthesis genes germacrene-D synthase (ClCG10G002820), limonene synthase (ClCG02G000830), and germacrene-D synthase (ClCG10G003700) were 128, 44, and 41 times, respectively, upregulated in the susceptible genotypes 10 dpi. After extending the inoculation period to 15 days, the expression levels of genes germacrene-D synthase (ClCG10G002820), limonene synthase (ClCG02G000830), and germacrene-D synthase (ClCG10G003700) increased even further, showing elevations of 315, 28, and 16 times, respectively. The expression level of these genes did not decrease significantly in the resistant variety.

### Validation of RNA-seq data by qRT−PCR

**Figure 5:**
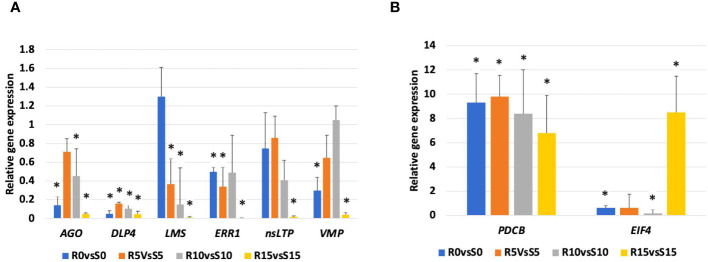
qRT-PCR validation of the relative expression levels of eight selected DEGs. Expression levels of argonaute (*AGO*), dicer like protein 4 (*DLP4*), limonene synthase (*LMS*), ethylene-responsive proteinase inhibitor 1 (*ERPI*), non specific lipid transfer protein (nsLTP), viral movement protein (*VMP*), plasmodesmata callose binding protein (*PDCB*), and eukaryotic initiation factor 4 (*EIF4*) genes in 392291-VDR (VDR) genotype compared to Crimson Sweet (CS) at 0, 5, 10, and 15 dpi. Subfigures **(A, B)** respectively represent the downregulation and upregulation of the genes, respectively. Actin was used as an internal control in qRT-PCR and data are represented as the mean of three biological replicates. Asterisks indicate statistically significant differences compared to control (student's t-test): *p<0.05.

To validate the RNA-Seq data, eight important DEGs were selected for gene expression analysis via qRT−PCR (**Figures 5A, B**). Similar to transcriptome data, *AGO, DLP4, LMS, ERP1, nsLTP*, and *VMP* genes were downregulated in the 392291-VDR genotype compared to Crimson Sweet, as observed by qRT-PCR. *PDCB* and *EIF4* genes were overexpressed in the 392291-VDR genotype compared to Crimson Sweet, which is congruent with transcriptome data. Overall, the qRT-PCR results validated the expression pattern of selected DEGs observed in RNA-Seq data.

## Discussion

A transcriptomic analysis was performed to explore the differences in gene expression between resistant and susceptible genotypes before and after inoculation with SqVYV to understand the mechanism of virus resistance in watermelon. This study revealed significant variations in the expression levels of several genes associated with diverse biological processes and some of them potentially associated with disease resistance. Our study revealed that suppression of virus replication and plasmodesmata callose deposition mechanism were activated in the resistant genotype, suggesting a possible role of these mechanisms in SqVYV resistance.

The *PDCB* gene was highly expressed in the resistant genotype compared to the susceptible genotype before and after SqVYV inoculation. In the susceptible genotype, the expression level of *PDCB* sharply decreased, while the resistant genotype maintained the gene expression level at 5 and 10 dpi. Callose is a polysaccharide made up primarily of β-1,3-glucan chains. In plants, it plays important roles in the defense against biotic stresses, including viral infections. Callose deposition at plasmodesmata inhibits the cell-to-cell movement of the viruses in plants and increases virus resistance (Li et al., 2012; Wang et al., 2021; Zhang et al., 2022). *PDCB*-overexpressing transgenic lines showed increased callose deposition and reduced symplastic transport in *Arabidopsis* (Simpson et al., 2009; Maule et al., 2013). In a genome-wide association study, the presence of genomic regions associated with SqVYV resistance in watermelon has been identified on chromosome 5, and the *PDCB* gene is situated on the same chromosome (Kousik, 2022). Callose deposition serves as an indicator of a plant’s resistance to a virus. β-1,3-glucanases play a key role in callose metabolism, a vital part of plasmodesmata, commonly linked with the movement of viruses (Iglesias and Meins, 2000; Li et al., 2012; Wang et al., 2021; Zhang et al., 2022). Viruses control the activity of β-1,3-glucanase and cause the callose in plasmodesmata to break down, enlarging their size-exclusion limit. This enlargement facilitates the movement of viral particles through them. Generally, (+) RNA viruses manipulate β-1,3-glucanases for the successful invasion and transportation in the plants (Iglesias and Meins, 2000). β-1,3-glucanase-deficient tobacco plants show resistance to tobacco mosaic virus, and these plants inhibit viral movement between cells (Iglesias and Meins, 2000). Overall, these results suggest the involvement of callose deposition in SqVYV resistance and the role of β-1,3-glucanase in susceptibility in watermelon.

Plants activate the RNA silencing defense mechanism in response to viral infection (Vance and Vaucheret, 2001; Wang et al., 2012; Guerra et al., 2019; Lopez-Gomollon and Baulcombe, 2022). In our study, five DEGs associated with the RNA silencing defense mechanism were differentially expressed between the resistant and susceptible genotypes after inoculation. These DEGs include eukaryotic initiation factors (ClCG11G014680, ClCG06G001240), translin (ClCG10G020230), Dicer like protein 4 (ClCG06G012100), RNA-dependent RNA polymerase (ClCG01G006600, ClCG01G006450), and Argonaute (ClCG01G014010). Eukaryotic initiation factor 4 (ClCG11G014680), Argonaute (ClCG01G014010) and Dicer like protein 4 (ClCG06G012100) were overexpressed in the susceptible genotype after inoculation but remained consistently expressed in the resistant line. In contrast, eukaryotic initiation factor 3 (ClCG06G001240) and translin (ClCG10G020230) were downregulated in the susceptible genotype after inoculation but exhibited consistent expression in the resistant line. Dicer like protein play important role viral resistant in plants (Moissiard and Voinnet, 2006; Andika et al., 2015; De-Souza et al, 2019). It regulates cauliflower mosaic virus resistance in Arabidopsis via RNAi (Moissiard and Voinnet, 2006). Furthermore, differential expression of the Dicer-like proteins contributes to antiviral defenses against potato virus X in tobacco (Andika et al., 2015). *eIF4* is a complex involved in the initiation of translation. Modifying eIF4, particularly *eIF4E*, has shown promise in developing virus-resistant plants (De-Souza et al, 2019; Le et al., 2022; Lucioli et al., 2022). CRISPR/Cas9 assisted mutagenesis in *eIF4E* gene enhance PVY resistance in tobacco (Le et al., 2022). Similarly, genome editing of *eIF4E1* gene increase PVY Resistance in eggplant (Lucioli et al., 2022). *AGO* gene family plays an important role in RNA silencing and regulation of disease resistance in plants. *AGO* proteins are key components of the RNA-induced silencing complex, which mediates gene silencing through mechanisms such as microRNA (miRNA) and small interfering RNA (siRNA) pathways. Recent studies have demonstrated the role of AGO genes in plant resistance to viruses (Kamitani et al., 2016; Zheng et al., 2017; Zanardo et al., 2019; Xu et al., 2022). The expression levels of two RNA-dependent RNA polymerase (RDR) genes (ClCG01G006600 and ClCG01G006450) increased in both genotypes after inoculation, but in the susceptible genotype, these genes were significantly overexpressed. The differential expression of *RDR* related genes in plant defense against viruses has been reported in various studies (Kamitani et al., 2016; Zanardo et al., 2019; Xu et al., 2022). *RDR1* mutation caused decreased resistance in tobacco plants to tobacco mosaic virus (TMV) (Vaucheret, 2006).

Two ribosome-inactivating proteins (ClCG08G004120, ClCG08G004130) were overexpressed in the resistant genotype after inoculation, while these genes were downregulated in the susceptible genotype. Ribosome-inactivating proteins enhance virus resistance in plants by arresting virus protein synthesis during translation (Zhu et al., 2018). Overexpression of ribosome-inactivating proteins in transgenic plants have been associated with resistance to various viruses such as cucumber mosaic virus (CMV), potato virus Y, potato virus X, turnip mosaic virus, potato leafroll virus, and TMV (Zhu et al., 2018). In addition, exogenous application of mirabilis antiviral protein, a type I ribosome-inactivating proteins showed strong resistance against CMV, and cucumber green mottle mosaic virus (CGMMV) in tobacco (Kubo et al., 1990). Furthermore, a recombinant ribosome-inactivating proteins showed strong resistance against TMV (Choudhary et al., 2008). Exogenous application of type I ribosome-inactivating proteins, pokeweed antiviral protein (PAP), enhance zucchini yellow mosaic virus (ZYMV) resistance in squash plants (Sipahioglu et al., 2017). Similar to our study, eight ribosome-inactivating proteins including ClCG08G004120 and ClCG08G004130 were overexpressed in resistant genotype compared to susceptible in response to potyvirus infection in watermelon, further suggesting role of ribosome-inactivating proteins in virus resistance in watermelon (Chanda et al., 2022). The differential expression of genes involved in virus genome replication and protein synthesis inhibition suggests the role of these mechanisms in SqVYV resistance.

There were significant differences in the expression of ET, CT, GA, JA, and SA hormone- related genes in both genotypes after SqVYV infection. Plant hormones play an important role in the growth and development of plants, some of which are essential for plant resistance against pathogens. Salicylic acid, JA, and ET play an important role in disease resistance. These hormone pathways are interconnected and crosstalk with each other (Denancé et al., 2013; Yang et al., 2013; Kaniganti et al., 2022). Ethylene-responsive proteinase inhibitor 1 (ClCG07G000740) and Ethylene insensitive like 3 (ClCG00G002220) were upregulated in the susceptible genotype after inoculation relative to the resistant genotype. Ethylene-responsive transcription factor (ClCG05G009970) was upregulated in the resistant genotype after inoculation. Plants viruses are known to manipulate the ET response pathway in plants by suppressing a plant’s defense mechanism including RNA silencing (Endres et al., 2010; Zhao et al., 2017). Viruses hijack ethylene biosynthesis and response pathways in the susceptible genotype for successful invasion. The involvement of ethylene hormone in plant-virus interaction has been reported in previous transcriptomic studies (Choi et al., 2015; Kalapos et al., 2021; Chen et al., 2022). Therefore, we hypothesized that ethylene-responsive proteinase inhibitor 1 (ClCG07G000740) and ethylene insensitive like 3 are required for susceptible reaction, and ethylene-responsive transcription factor is required for plant resistance against SqVYV. Salicylate 3-hydroxylase (ClCG01G001350) was upregulated in the susceptible genotype, while salicylic acid-binding protein (ClCG09G017610) was upregulated in the resistant variety after inoculation. Salicylic acid is a key hormone that plays an important role in plant defense against viruses (Denancé et al., 2013; Yang et al., 2013; Slavokhotova et al., 2021). Differential expression of SA biosynthesis pathway genes after virus infection has also been observed in in other studies (Fernando Gil et al., 2020; Slavokhotova et al., 2021; Sáez et al., 2022). Several JA biosynthesis pathway genes were differentially expressed between the resistant and susceptible genotypes after SqVYV inoculation. Jasmonic acid-amido synthetase (ClCG02G017430) was downregulated in the susceptible variety, while jasmonate O-methyltransferase (ClCG05G018780) was downregulated in both genotypes at 5 dpi. Jasmonate ZIM domain-containing protein 10 (ClCG06G001800) was upregulated in the susceptible variety at 10 and 15 dpi. Differential expression of JA biosynthesis pathway genes in response to virus infection has also been observed in other studies (Fernando Gil et al., 2020; Slavokhotova et al., 2021; Sáez et al., 2022). The dual positive and negative roles of JA in plant defense against viruses have been reported in various studies (Zhao and Li, 2021). Additionally, genes associated with the biosynthesis and response to GA, cytokinin, and auxin showed differential expression. This could be attributed to interactions between these hormones and ET, SA, and JA. Overall, our results suggest possible roles for ET, SA, and JA hormones in watermelon resistance against SqVYV infection.

Proteinase inhibitors, *LTPs*, *ACD*, *ABC* transporters, ubiquitin, and histones are important components of plant defense (Patkar and Chattoo, 2006; Smith and Boyko, 2007; Huang et al., 2016; Amador et al., 2021; Missaoui et al., 2022; Shang et al., 2022). Three nsLTPs were downregulated in the susceptible genotype after inoculations, while their expression in the resistant genotype was unchanged. LTPs are small, cysteine-rich proteins that promote plant defense against pathogens, including viruses (Patkar and Chattoo, 2006; Huang et al., 2016; Amador et al., 2021; Missaoui et al., 2022; Shang et al., 2022). The downregulation of nsLTP genes in the susceptible genotype after inoculation suggested that SqVYV overcomes this layer of watermelon resistance. Proteinase inhibitors in plants are defense proteins that are traditionally known for protecting against herbivores. However, they also play a role in defense against viruses by inhibiting viral proteases, thereby potentially reducing viral replication (Gutierrez-Campos et al., 1999; Nair et al., 2023; Soleimani et al., 2023). Viruses need to overcome this layer of defense to cause disease in plants. In our study, the susceptible genotype had a significant reduction in the expression of proteinase inhibitor genes, and the resistant genotype maintained the expression of these genes, suggesting the role of proteinase inhibitor genes in SqVYV resistance in watermelon. Some of these proteinase genes overexpressed in the susceptible genotype after inoculation. These proteinases might be negative regulators of SqVYV resistance in watermelon. Furthermore, ABC transporters, ubiquitin, and histones play crucial roles in plant disease resistance. ABC transporters help in defense by transporting antimicrobial compounds and detoxifying pathogen-produced toxins (Devanna et al., 2021; Panigrahi et al., 2021; Banasiak and Jasiński, 2022; Qu et al., 2023). The ubiquitin−proteasome system regulates plant immunity by targeting specific defense proteins for degradation, although some pathogens manipulate this system to weaken defenses (Panigrahi et al., 2021). Moreover, histone modifications, such as acetylation, modulate the expression of defense-related genes, either by amplifying or suppressing plant defense responses based on the nature of the modification (Panigrahi et al., 2021; Qu et al., 2023). The differential expression of ABC transporters, ubiquitin, and histone-related genes suggested their role in SqVYV resistance in watermelon.

The cell wall of plants is a crucial structural component that provides support and defense against environmental stresses and pathogens. Virus infections in plants can lead to the degradation of the cell wall, increased plasmodesmata permeability, and alterations in cell wall components, which facilitate the spread of the virus and the manifestation of symptoms. However, in resistant plant genotypes, these changes in the cell wall are minimized or prevented, maintaining cell wall stability and hindering the virus’s ability to spread (Li et al., 2017; Kozieł et al., 2021). Watermelon fruits infected by Cucumber green mottle mosaic virus showed differential expression of pectin, cellulose, and lignin regulated genes (Li et al., 2017). Transcriptional analysis of South African cassava mosaic virus-infected susceptible and tolerant landraces of cassava showed upregulation of the cell wall related genes after infection (Allie et al., 2014). Our transcriptome analysis revealed significant differential expression of specific genes related to pectin metabolism and modification, cellulose synthesis, cell growth and development, xenobiotic metabolism, lignin biosynthesis, and defense against chitin-containing pathogens. Although, more DEGs were found in susceptible genotype compared to resistant suggest more cell wall degradation in susceptible genotype after inculcation. The downregulation of genes involved in pectin metabolism and modification, cellulose synthesis, and cell growth and development suggested potential alterations in cell wall integrity, defense responses, and growth processes in response to SqVYV infection. Similar to our study, differential expression of cellulose, hemicellulose, and pectin related genes have been reported in Arabidopsis thaliana Infected by Tomato spotted wilt virus (Xu et al., 2020, 2022). Similarly, comparative transcriptome analysis demonstrates the role of lignin synthesis genes in regal lily against cucumber mosaic virus and tobacco mosaic virus (Sun et al., 2019). Additionally, the downregulation of genes associated with lignin biosynthesis and defense against chitin-containing pathogens implies potential modulation of stress responses, xenobiotic metabolism, lignin production, and defense mechanisms against chitin-containing pathogens in SqVYV resistance. Terpene biosynthesis pathway genes were highly overexpressed in the susceptible genotype after SqVYV inoculation. Further research is needed to elucidate the role of terpenoids in SqVYV infection of watermelon.

Our transcriptome data provided a basis for a comprehensive understanding of the gene expression profiles of resistant and susceptible watermelon varieties at different stages of SqVYV infection and provided insight into the SqVYV resistance mechanism. Our analysis revealed significant differential expression of genes involved in pathogenesis-related processes, including ubiquitin-mediated proteolysis, histone modifications, and antiviral RNA interference (RNAi) pathways. The results highlighted the important genes involved in the ETH, JA, and SA signaling pathways and related to the response to SqVYV infection that were differentially expressed between the susceptible and resistant varieties. These findings will be helpful for understanding the molecular mechanisms of SqVYV resistance in watermelon.

## Data availability statement

The data presented in the study are deposited in the NCBI repository, accession number PRJNA1086032.

## Author contributions

RK: Writing – review & editing, Writing – original draft, Visualization, Validation, Software, Investigation, Formal analysis. BC: Writing – review & editing, Methodology. SA: Writing – review & editing, Methodology, Conceptualization. CK: Writing – review & editing, Visualization, Supervision, Resources, Project administration, Investigation, Funding acquisition, Conceptualization.
